# Efficacy of sarolaner in Simparica^®^ (sarolaner) chewables and Simparica Trio^®^ (sarolaner, moxidectin, and pyrantel chewable tablets) against two US strains of *Haemaphysalis longicornis*

**DOI:** 10.1186/s13071-025-07202-2

**Published:** 2026-01-11

**Authors:** Jessica Rodriguez, Shelby Jones, Lucas Taylor, Jody DeMarco, Keith Baker, Melanie Myers

**Affiliations:** Zoetis, Veterinary Medicine Research and Development, Kalamazoo, MI USA

**Keywords:** *Haemaphysalis longicornis*, Asian longhorned tick, Dogs, Ectoparasiticide, Isoxazoline, Efficacy, Sarolaner

## Abstract

**Background:**

*Haemaphysalis longicornis* (Asian longhorned tick) is an invasive species now established in the Northeastern and Mid-Atlantic USA. It feeds on mammalian wildlife, livestock, birds, cats, dogs, and humans. Simparica^®^ and Simparica Trio^®^ contain sarolaner, a drug in the isoxazoline class, with activity against fleas, ticks, and mites.

**Methods:**

Two laboratory studies were conducted using 30 dogs each, randomized into three groups (*n* = 10/group): placebo (Pet Tabs^®^), Simparica Trio (minimum dose: 1.2 mg/kg sarolaner, 24 µg/kg moxidectin, 5 mg/kg pyrantel, as pamoate salt), and Simparica (minimum dose: 2.0 mg/kg sarolaner). Treatments were administered once orally on Day 0 according to the approved commercial dosing directions. Each dog was infested with 50 (± 5) unfed viable adult female *H. longicornis* on Days -2, 7, 14, 21, 30, 37, 49, and 63, and ticks were counted with removal and categorization at 48 h after treatment and each subsequent infestation. Ectoparasitic efficacy was calculated on the basis of the reduction in arithmetic mean of live and dead tick counts in each of the treated groups versus the untreated control group for every time point post infestation.

**Results:**

Adequate challenge was demonstrated in both studies on the basis of live tick counts at each time point. For all sarolaner-treated groups, mean live counts were significantly (*P* ≤ 0.0005) lower than those for the placebo at all time points. For Simparica, in Study 1, the percentage reductions were 100% for all time points up to Day 39. On Days 51 and 65, the percentage reductions were 98.9% and 82.4%, respectively. In Study 2, reductions were 99.7–100% up to Day 65. For Simparica Trio, in Study 1, percentage reductions were 100% up to Day 51. On Day 65, the percentage reduction was 78.4%. In Study 2, reductions were 99.6–100% up to Day 39 and 97.6% and 94.1% on Days 51 and 65, respectively.

**Conclusions:**

Results from these controlled studies demonstrated high efficacy (78.4–100%) of Simparica and Simparica Trio in reducing existing and subsequent infestations of *H. longicornis* within 48 h for up to 65 days post treatment.

**Graphical Abstract:**

**Figure:**
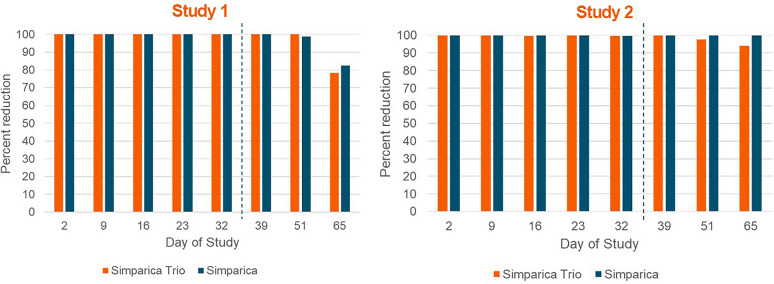

## Background

*Haemaphysalis longicornis*, also referred to as the Asian longhorned tick, is a tick species that originated from southeast Asia and over time has established in other regions, including Australia, New Zealand, and more recently in several states in the eastern half of the USA [1]. This tick feeds on multiple hosts from birds and mammalian wildlife to livestock, cats, dogs, and humans [2]. In addition to sexual reproduction, these ticks also can reproduce asexually by parthenogenesis. It also is responsible for transmitting several pathogens of veterinary and medical importance, including *Theileria orientalis* Ikeda genotype in cattle (bovine theileriosis) in North America, Asia, and Oceania and Dabie bandavirus in humans (severe fever with thrombocytopenia syndrome) in Asia [1].

The Asian longhorned ticks that have established in the USA are parthenogenetic and likely to have had at least four separate introductions into the USA, with East Asian origins through dog sources likely given the lack of biosecurity measures on dogs arriving in the USA from low rabies-risk countries [3]. In general, in regions studied in the Northeastern USA, these ticks are found from early spring through the fall, with nymphs peaking in the spring, adults in the summer, and larvae in the fall; however, the season may be shorter in the Midwestern USA [4]. Laboratory studies using *H. longicornis* ticks from North America have demonstrated their ability to transmit some pathogens, such as *Rickettsia rickettsii*, Heartland virus, and Powassan virus [5–7]. As of April 2025, dogs were the most frequently reported host infested with *H. longicornis* (179 cases), according to the US Department of Agriculture [8].

Simparica^®^ and Simparica Trio^®^ contain sarolaner, a drug in the isoxazoline class, which exhibits activity against fleas, ticks, and mites [9–11]. Both products have demonstrated robust efficacy against *H. longicornis* ticks of Japanese origin for up to 5 weeks post treatment [12, 13]. Here, we evaluated the efficacy of these two products in dogs against two US strains of *H. longicornis*.

## Methods

### General study design and animal management

Two negative-controlled laboratory studies were conducted. The study design and procedures were conducted in accordance with the World Association for the Advancement of Veterinary Parasitology guidelines for evaluating the efficacy of parasiticides for the treatment, prevention, and control of flea and tick infestations in dogs and cats [14]. In Study 1, 36 male and female purpose-bred beagle dogs were acclimatized to the study site 14 days prior to treatment. The 30 dogs with the highest host-suitability tick counts were selected for the study. The selected study dogs included 14 males and 16 females, ranged from 40 to 61 weeks of age, and weighed 5.8–9.9 kg. In Study 2, 34 male and female purpose-bred beagles, beagle crosses, and mongrel dogs were acclimatized to the study site 14 days prior to treatment. The 30 dogs with the highest host-suitability tick counts were selected for the study. The selected study dogs included 16 males and 14 females. The dogs ranged from 7 to 79 months of age and weighed 10.9–21.4 kg. The dogs were identified by uniquely numbered ear tattoos and housed individually in indoor enclosures in tick-proof facility that conformed to accepted animal welfare guidelines and ensured no direct contact between dogs. Prior to infestation on Day −7, all dogs were examined to ensure they were free of ticks. On Day −7, each of the dogs were infested with approximately 50 (± 5) viable, female unfed adult *H. longicornis* ticks. On Day −5 (48 ± 2 h after infestation on Day −7), the number of live attached ticks present on each dog was counted, to determine host suitability, and removed but not assessed for engorgement status. The 30 dogs with the highest host-suitability tick counts were allocated according to a randomized complete block design to one of three treatment groups in each block (total of ten dogs per treatment group): T01, placebo treatment group; T02, Simparica Trio-treated group (sarolaner, moxidectin, and pyrantel); and T03, Simparica-treated group (sarolaner).

For treatment groups T02 and T03, tablets were administered on the basis of label directions: Simparica Trio (T02) minimum dose: 1.2 mg/kg sarolaner, 24 µg/kg moxidectin, 5 mg/kg pyrantel; and Simparica (T03) minimum dose: 2 mg/kg. The tablet(s) were administered orally, while food was withheld overnight (at least 12 h) prior to treatment administration, and dogs were not fed again until at least 4 h post treatment. On Day 0 prior to treatment, 1 h, 3 h, 6 h, and 24 h after dosing, all dogs were assessed for general health. Throughout the study (Day −14 to Day 65), general health was observed by appropriately trained personnel at least twice daily. All persons who made observations or performed general care for the dogs were masked to treatment assignment. Both studies were conducted in compliance with the CVM Guidance for Industry #85, Good Clinical Practice, VICH GL9. Animal management practices at the study sites complied with the US Department of Agriculture (USDA) Animal Welfare Act requirements.

### Tick infestations and counts

For Study 1, the *H. longicornis* isolate was obtained from the Centers for Disease Control and Prevention (CDC; Atlanta, GA) in January 2020. The isolate was initiated from ticks originating from New York, USA, which were collected from the field on 9 July 2018. For Study 2, the *H. longicornis* isolate was obtained from the Utrecht Centre for Tick-borne Diseases (Utrecht, Netherlands) in January 2019. The isolate was initiated from ticks originating from Virginia, USA, which were collected from the field in October 2018.

On Days −2, 7, 14, 21, 30, 37, 49, and 63, each dog was infested with 50 (± 5) viable, unfed adult female *H. longicornis* ticks. Ticks were applied directly to each dog. Three vials from each batch were checked to demonstrate that the vials prepared by laboratory standard operating procedure (SOP) contained the appropriate number of viable, unfed female adult ticks. Prior to all tick infestations, all dogs were sedated. In Study 1, dogs were sedated with DEXDOMITOR^®^ (dexmedetomidine hydrochloride, Orian Corporation, Espoo, Finland) or DEXSED^™^ (dexmedetomidine hydrochloride, Aspen Veterinary Resources, Liberty, MO, USA) by intramuscular injection at a dose of 0.02 mg/kg. In Study 2, dogs were sedated with DOMITOR^®^ (medetomidine hydrochloride, Zoetis, South Africa) by intramuscular injection at a dose of 0.06 mg/kg. Dogs were confined to individual infestation chambers for approximately 2 h to enhance tick attachment. If ticks did not fully enter the fur after 2 h, the infestation period could be extended up to approximately 4 h.

All persons conducting post-treatment tick infestations and post-treatment tick counts were masked to treatment assignment. Tick counts were conducted in pen order at 48 (± 2) h after Day 0 treatment and 48 (± 2) h after each subsequent infestation on Days 7, 14, 21, 30, 37, 49, and 63. During tick counts, the number of live and dead (attached and free) ticks present on each dog were counted, removed, and categorized according to engorgement status [14]. The following guidelines were followed:

- To facilitate counting, mechanical counters were used to assist in quantifying the ticks.
- The dog was placed on a table, its identity confirmed, and the start time was recorded.
- Tick counts were made first by a general examination to identify any ticks that were readily visible.
- To further examine an animal for ticks, the hair was pushed against its natural nap. The skin and any ticks were exposed using this process. Beginning at the head area, the examiner proceeded to cover all areas of the animal that could be examined from the top. When the top areas of the animal were examined completely, the animal was gently turned on its back. The underside was examined by pushing the hair against its natural nap to expose the skin and ticks.
- After manual inspection, an extra-fine-toothed comb was used to comb the animal to remove any otherwise missed ticks. Each dog was examined for at least 10 min. If ticks were encountered in the last minute, combing was continued in 1-min increments until no ticks were encountered.
- The surface of the workspace was cleaned thoroughly with appropriate cleaner between each animal. Protective clothing was changed between each animal.
- After removal, counting and categorization of the ticks, the ticks were placed in 70% isopropyl alcohol and discarded.

### Statistical analysis

Data were summarized and analyzed using SAS^®^ software, Version 9.4 (SAS Institute Inc., Cary, NC, USA). The experimental unit for treatment was the individual dog and the primary efficacy endpoint was the live tick count. Data for post-treatment live (free + attached) and dead tick counts were summarized with arithmetic means and least squares means by treatment group and time point. Live tick percentage effectiveness of the investigational veterinary product (IVP)-treated group with respect to the placebo-treated group was calculated at each time point using the formula [(*C* − *T*) / *C*] × 100, where *C* is the arithmetic mean of live tick counts for the placebo-treated group and *T* is the arithmetic mean of live tick counts for the IVP-treated group. Mean live tick counts at each time point were compared using a mixed linear model for live tick counts with fixed effect of treatment and random effects of block and error. The study had two primary hypothesis tests: (1) comparison between Simparica and control to support the effectiveness claim for Simparica and (2) comparison between Simparica Trio and control to support the effectiveness claim for Simparica Trio. A multiplicity adjustment was made to control the overall type I error rate at the 0.05 level (two-sided), using the Dunnett multiple comparison adjustment method. Efficacy endpoints and treatment differences were assessed at each time point through Day 65; however, the criteria for the control and treatment indications only needed to be met through Day 32 for the IVP to be considered effective.

## Results

### Health observations

In Study 1, there were no mortalities or product-related abnormal health events. In Study 2, one dog had a seizure, described as mild twitching and foam at the mouth, 16 days after administration with Simparica Trio (T02). This abnormal health event resolved without treatment after 2 minutes.

### Study 1

The actual dose range of sarolaner in T02 (Simparica Trio) was 1.2–1.9 mg/kg and in T03 (Simparica) was 2.1–3.3 mg/kg. The 30 dogs selected with the highest live attached tick counts ranged from 12 to 38 ticks per dog (24–76% retention). For efficacy assessments, a summary of live tick counts with means, ranges, percentage reduction, and statistical comparisons is presented in Table 1 and a summary of dead tick counts with means and ranges is presented in Table 2. All placebo-treated dogs maintained an infestation of *H. longicornis* at each time point; however, fewer than 12 live ticks were recovered from eight of the placebo-treated dogs at some time points throughout the study (Table 1). Infestations were considered adequate, as all placebo-treated dogs held live ticks, at least six control dogs were adequately infested (≥ 12 live ticks) at each time point, and mean counts were greater than 12 (range 13.1–31.4). The mean dead tick counts for the Simparica Trio- and Simparica-treated groups were higher than that of the placebo-treated group on all days. Percentage reductions in arithmetic mean live tick counts compared with placebo for the Simparica Trio-treated group were 100% on Days 2, 9,16, 23, 32, 39, and 51. On Day 65, the percentage reduction was 78.4%. Mean live counts were significantly (*P* ≤ 0.0005) lower than those for the placebo at all time points. Percentage reductions in arithmetic mean live tick counts compared with placebo for the Simparica-treated group were 100% on Days 2, 9, 16, 23, 32, and 39. On Days 51 and 65, the percentage reductions were 98.9% and 82.4%, respectively. Mean live counts were significantly (*P* ≤ 0.0003) lower than those for the placebo at all time points. Efficacy (≥ 90% reduction in live ticks) for both sarolaner-treated groups was achieved through Day 51.

**Table 1:** Summary of live tick counts with means, percent reduction, and statistical comparisons for dogs treated orally with Simparica Trio and Simparica in Study 1

Treatment	Animal	Day of study							
		2	9	16	23	32	39	51	65
T01 placebo (Pet-Tabs^®^)	4800266	13	16	43	44	38	22	14	8
	4820292	12	18	14	31	37	27	15	3
	4831587	18	13	43	40	32	30	15	22
	4839383	19	16	35	31	31	26	22	20
	4842104	18	19	24	6	14	23	16	14
	4860528	14	4	33	17	20	20	1	6
	4862181	10	7	30	27	30	22	21	14
	4862377	3	40	48	34	20	44	33	12
	4880286	5	11	21	37	30	27	24	28
	4880413	19	6	23	30	19	18	25	21
	Range	3–19	4–40	14–48	6–44	14–38	18–44	1–33	3–28
	Arithmetic mean	13.1	15.0	31.4	29.7	27.1	25.9	18.6	14.8
	LS mean	13.1	15.0	31.4	29.7	27.1	25.9	18.6	14.8
T02 Simparica Trio^®^	4795424	0	0	0	0	0	0	0	0
	4802838	0	0	0	0	0	0	0	7
	4835817	0	0	0	0	0	0	0	1
	4839693	0	0	0	0	0	0	0	2
	4843097	0	0	0	0	0	0	0	13
	4856539	0	0	0	0	0	0	0	0
	4859864	0	0	0	0	0	0	0	0
	4866772	0	0	0	0	0	0	0	0
	4892403	0	0	0	0	0	0	0	8
	4906757	0	0	0	0	0	0	0	1
	Range	0–0	0–0	0–0	0–0	0–0	0–0	0–0	0–13
	Arithmetic mean	0.0	0.0	0.0	0.0	0.0	0.0	0.0	3.2
	% reduction (arithmetic mean)	100.0	100.0	100.0	100.0	100.0	100.0	100.0	78.4
	LS mean	0.0	0.0	0.0	0.0	0.0	0.0	0.0	3.2
	% reduction (LS mean)	100.0	100.0	100.0	100.0	100.0	100.0	100.0	78.4
Simparica Trio^®^ versus placebo	*P*-value	< 0.0001	< 0.0001	< 0.0001	< 0.0001	< 0.0001	< 0.0001	< 0.0001	0.0005
T03 Simparica^®^	4823142	0	0	0	0	0	0	2	14
	4824319	0	0	0	0	0	0	0	1
	4833199	0	0	0	0	0	0	0	0
	4841035	0	0	0	0	0	0	0	0
	4853246	0	0	0	0	0	0	0	0
	4855257	0	0	0	0	0	0	0	0
	4876173	0	0	0	0	0	0	0	0
	4877854	0	0	0	0	0	0	0	0
	4880812	0	0	0	0	0	0	0	0
	4905092	0	0	0	0	0	0	0	11
	Range	0–0	0–0	0–0	0–0	0–0	0–0	0–2	0–14
	Arithmetic mean	0.0	0.0	0.0	0.0	0.0	0.0	0.2	2.6
	% reduction (arithmetic mean)	100.0	100.0	100.0	100.0	100.0	100.0	98.9	82.4
	LS mean	0.0	0.0	0.0	0.0	0.0	0.0	0.2	2.6
	% reduction (LS mean)	100.0	100.0	100.0	100.0	100.0	100.0	98.9	82.4
Simparica^®^ versus placebo	*P*-value	< 0.0001	< 0.0001	< 0.0001	< 0.0001	< 0.0001	< 0.0001	< 0.0001	0.0003

**Table 2:** Dead tick summary statistics—by day of study and treatment for dogs treated orally with Simparica Trio and Simparica in Study 1

Day of study	Treatment	Number of animals	Arithmetic mean	Standard deviation	Minimum	Maximum
2	T01 (Pet-Tabs^®^)	10	0.1	0.3	0	1
	T02 Simparica Trio^®^	10	0.7	1.3	0	4
	T03 Simparica^®^	10	2.4	2.9	0	7
9	T01 (Pet-Tabs^®^)	10	0.1	0.3	0	1
	T02 Simparica Trio^®^	10	2.2	2.1	0	7
	T03 Simparica^®^	10	2.4	2.1	0	6
16	T01 (Pet-Tabs^®^)	10	0.1	0.3	0	1
	T02 Simparica Trio^®^	10	1.4	2.5	0	6
	T03 Simparica^®^	10	1.5	3.0	0	9
23	T01 (Pet-Tabs^®^)	10	0.5	1.0	0	3
	T02 Simparica Trio^®^	10	3.3	2.1	0	6
	T03 Simparica^®^	10	3.7	3.7	0	12
32	T01 (Pet-Tabs^®^)	10	0.0	0.0	0	0
	T02 Simparica Trio^®^	10	2.1	2.7	0	9
	T03 Simparica^®^	10	4.2	2.8	0	8
39	T01 (Pet-Tabs^®^)	10	0.1	0.3	0	1
	T02 Simparica Trio^®^	10	2.1	2.4	0	6
	T03 Simparica^®^	10	1.1	1.7	0	5
51	T01 (Pet-Tabs^®^)	10	0.1	0.3	0	1
	T02 Simparica Trio^®^	10	1.9	2.2	0	7
	T03 Simparica^®^	10	2.1	1.6	0	5
65	T01 (Pet-Tabs^®^)	10	0.1	0.3	0	1
	T02 Simparica Trio^®^	10	4.3	5.2	0	18
	T03 Simparica^®^	10	1.1	1.0	0	3

### Study 2

The actual dose range of sarolaner in T02 (Simparica Trio) was 1.2–2.4 mg/kg and in T03 (Simparica) was 2.0–3.7 mg/kg. The 30 dogs selected with the highest live attached tick counts ranged from 19 to 46 live ticks per dog (38–92% retention). For efficacy assessments, a summary of live tick counts with means, ranges, percentage reduction, and statistical comparisons is presented in Table 3 and a summary of dead tick counts with means and ranges is presented in Table 4. All placebo-treated dogs maintained an infestation of *H. longicornis* at each time point. However, fewer than 12 live ticks were recovered from five of the placebo-treated dogs on some time points throughout the study (Table 3). Infestations were considered adequate, as all placebo-treated dogs held live ticks, at least six control dogs were adequately infested (≥ 12 live ticks) at each time point, and mean counts were greater than 12 (range 15.2–31.5). The mean dead tick counts for the Simparica Trio- and Simparica-treated groups were higher than that of the placebo-treated group on all days except on Day 65. Percentage reductions in arithmetic mean live tick counts compared with placebo for the Simparica Trio-treated group were 100% on Days 2, 9, 23, and 39 and were 99.6%, 99.7%, 97.6%, and 94.1% on Days 16, 32, 51, and 65, respectively. Mean live counts were significantly (*P* < 0.0001) lower than those for the placebo at all time points. Percentage reductions in arithmetic mean live tick counts compared with placebo for the Simparica-treated group were 100% on Days 2, 9,16, 23, 39, 51, and 65 and were 99.7% on Day 32. Mean live counts were significantly (*P* < 0.0001) lower than those for the placebo at all time points. Efficacy (≥ 90% reduction in live ticks) for both sarolaner-treated groups was achieved through Day 65.

**Table 3:** Summary of live tick counts with means, percentage reduction, and statistical comparisons for dogs treated orally with Simparica Trio and Simparica in Study 2

Treatment	Animal	Day of study							
		2	9	16	23	32	39	51	65
T01 placebo (Pet-Tabs^®^)	86A B8F	36	21	26	32	36	31	28	28
	C6D D40	34	14	15	37	32	24	24	17
	C70 271	32	35	36	30	24	33	28	36
	CD3 88D	18	29	23	23	21	20	22	13
	CD3 9ED	31	7	21	20	24	21	24	15
	CD4 430	28	31	26	44	36	33	23	8
	CD8 FB9	31	34	30	27	27	20	23	17
	CD9 AE8	35	20	21	26	32	27	22	10
	CDA 0AA	33	25	25	30	25	36	30	8
	CDA 293	37	26	25	34	34	24	29	0
	Range	18–37	7–35	15–36	20–44	21–36	20–36	22–30	0–36
	Arithmetic mean	31.5	24.2	24.8	30.3	29.1	26.9	25.3	15.2
	LS mean	31.5	24.1	24.8	30.3	29.1	26.9	25.3	15.2
T02 Simparica Trio^®^	C6D DF3	0	0	0	0	0	0	0	0
	C6D FB0	0	0	0	0	0	0	0	0
	C6E A3E	0	0	1	0	1	0	0	9
	CD4 4C1	0	0	0	0	0	0	0	0
	CD5 906	0	0	0	0	0	0	0	0
	CD5 DEC	0	0	0	0	0	0	0	0
	CD6 B90	0	0	0	0	0	0	0	0
	CD7 B31	0	0	0	0	0	0	6	0
	CD8 3B2	0	0	0	0	0	0	0	0
	CD9 23C	0	0	0	0	0	0	0	0
	Range	0–0	0–0	0–1	0–0	0–1	0–0	0–6	0–9
	Arithmetic mean	0.0	0.0	0.1	0.0	0.1	0.0	0.6	0.9
	% reduction (arithmetic mean)	100.0	100.0	99.6	100.0	99.7	100.0	97.6	94.1
	LS rean	0.0	0.0	0.1	0.0	0.1	0.0	0.6	0.9
	% Reduction (LS mean)	100.0	100.0	99.7	100.0	99.6	100.0	97.6	94.3
Simparica Trio^®^ versus placebo	*P*-value	< 0.0001	< 0.0001	< 0.0001	< 0.0001	< 0.0001	< 0.0001	< 0.0001	< 0.0001
T03 Simparica^®^	B97 8D8	0	0	0	0	0	0	0	0
	C6E 21C	0	0	0	0	0	0	0	0
	C6E 3A2	0	0	0	0	0	0	0	0
	CD4 31F	0	0	0	0	0	0	0	0
	CD4 9F8	0	0	0	0	0	0	0	0
	CD5 18E	0	0	0	0	0	0	0	0
	CD5 B1C	0	0	0	0	1	0	0	0
	CD7 4D3	0	0	0	0	0	0	0	0
	CD8 5D2	0	0	0	0	0	0	0	0
	CD9 06B	0	0	0	0	0	0	0	0
	Range	0–0	0–0	0–0	0–0	0–1	0–0	0–0	0–0
	Arithmetic mean	0.0	0.0	0.0	0.0	0.1	0.0	0.0	0.0
	% reduction (arithmetic mean)	100.0	100.0	100.0	100.0	99.7	100.0	100.0	100.0
	LS mean	0.0	0.0	0.0	0.0	0.1	0.0	0.0	0.0
	% reduction (LS mean)	100.0	100.0	100.0	100.0	99.6	100.0	100.0	100.0
Simparica^®^ versus placebo	*P*-value	< 0.0001	< 0.0001	< 0.0001	< 0.0001	< 0.0001	< 0.0001	< 0.0001	< 0.0001

**Table 4:** Dead tick summary statistics—by day of study and treatment for dogs treated orally with Simparica Trio and Simparica in Study 2

Day of study	Treatment	Number of animals	Arithmetic mean	Standard deviation	Minimum	Maximum
2	T01 (Pet-Tabs^®^)	10	0.2	0.6	0	2
	T02 Simparica Trio^®^	10	8.2	4.5	4	17
	T03 Simparica^®^	10	7.3	2.6	3	10
9	T01 (Pet-Tabs^®^)	10	0.0	0.0	0	0
	T02 Simparica Trio^®^	10	3.0	2.1	0	6
	T03 Simparica^®^	10	2.5	1.4	0	5
16	T01 (Pet-Tabs^®^)	10	0.1	0.3	0	1
	T02 Simparica Trio^®^	10	2.1	2.3	0	8
	T03 Simparica^®^	10	1.4	1.1	0	3
23	T01 (Pet-Tabs^®^)	10	0.0	0.0	0	0
	T02 Simparica Trio^®^	10	1.3	1.7	0	5
	T03 Simparica^®^	10	1.1	1.2	0	4
32	T01 (Pet-Tabs^®^)	10	0.0	0.0	0	0
	T02 Simparica Trio^®^	10	1.7	2.2	0	7
	T03 Simparica^®^	10	1.1	1.2	0	4
39	T01 (Pet-Tabs^®^)	10	0.1	0.3	0	1
	T02 Simparica Trio^®^	10	1.5	1.4	0	5
	T03 Simparica^®^	10	1.4	1.9	0	6
51	T01 (Pet-Tabs^®^)	10	0.0	0.0	0	0
	T02 Simparica Trio^®^	10	1.0	1.6	0	5
	T03 Simparica^®^	10	1.0	0.9	0	2
65	T01 (Pet-Tabs^®^)	10	2.2	7.0	0	22
	T02 Simparica Trio^®^	10	1.8	1.8	0	5
	T03 Simparica^®^	10	1.5	1.0	0	3

## Discussion

These data demonstrate the robust efficacy of sarolaner in Simparica and Simparica Trio against existing and subsequent infestations of US-origin *H. longicornis* ticks in dogs for at least 30 days. The results from these studies are comparable with the results seen in previous studies assessing the efficacy of these products against *H. longicornis* originating from Japan [12, 13]. For Simparica Trio, efficacy based on arithmetic means in the studies described here ranged from 99.6% to 100% from Days 2 to 32, and in studies evaluating efficacy against Japanese-origin *H. longicornis* ticks, efficacy ranged from 97.9% to 100% from Days 2 to 35 [13]. For Simparica, efficacy based on arithmetic means in the studies described here ranged from 99.7% to 100% from Days 2 to 32, and in studies evaluating efficacy against Japanese-origin *H. longicornis* ticks, efficacy ranged from 95.9% to 100% from Days 2 to 35 [12].

In addition to efficacy against this invasive, and now established, tick in the USA, Simparica and Simparica Trio have demonstrated efficacy against five other tick species found in the USA that readily infest dogs: *Amblyomma americanum*, *Amblyomma maculatum*, *Dermacentor variabilis*, *Rhipicephalus sanguineus*, and *Ixodes scapularis* [10, 15]. Although the primary efficacy evaluation period was for the initial 30 days post product administration, the efficacy after Day 30 gives an indication of this tick species’ relative susceptibility to sarolaner compared with *Amblyomma* spp., previously determined to be the least sensitive US ticks to sarolaner in dogs [16]. In one previous laboratory study evaluating effectiveness of Simparica Trio at the minimum dose against *A. maculatum*, reduction in arithmetic mean live tick counts compared with placebo was ≥ 97% 48 h after infestation up to Day 35 [15]. In another study using a different strain of *A. maculatum*, percentage arithmetic mean live tick count reductions were ≥ 97.6% up to Day 21 infestations; however, efficacy 48 h after reinfestations on Days 28 and 35 were 90.4% and 76.3%, respectively [15]. In addition, in a dose-determination study for Simparica, using a sarolaner suspension dosed at 1.25 mg/kg, efficacy against *A. maculatum* 48 h after infestation on Day 43 was 68.3% [16]. In comparison, here in Study 2, when *H. longicornis* ticks were applied 63 days post treatment, the 48-h reduction in arithmetic mean live ticks was 100% for dogs administered Simparica (2.0–3.7 mg/kg sarolaner) and 94.1% for dogs administered Simparica Trio (1.2–2.4 mg/kg sarolaner). These data indicate that *H. longicornis* is more susceptible to sarolaner than *Amblyomma* spp.

Habitat suitability models predict that *H. longicornis* will extend beyond its currently established area in the eastern half of the USA. Models predict that the tick will be able to establish in the Midwest and Pacific Northwest of the USA and portions of the southeast and southwestern regions of Canada [17]. Effective control measures for this tick in the environment and protective measures for potential hosts should be implemented as appropriate. As the travel of humans with their pet dogs is mainstay, it is important to ensure that proper parasite control is used to minimize the risk of the movement of parasites and of acquiring new parasitic infections/infestations in new environments.

## Conclusions

Results from these controlled studies demonstrated high efficacy (78.4–100%) of a single dose of Simparica or Simparica Trio in reducing existing and subsequent infestations of *H. longicornis* within 48 h for up to 65 days post treatment.

## Data Availability

The dataset supporting the conclusions of this article is included within the article.

## Abbreviations

CDC: Centers for Disease Control and Prevention
CVM: Center for Veterinary Medicine
IVP: Investigational Veterinary Product
SOP: Standard operating procedure
USDA: US Department of Agriculture
VICH GL9: Veterinary International Conference on Harmonization Guideline 9
